# A feed-forward loop between Toll/NF-κB and Rac1 promotes epithelial to mesenchymal transition of Ras-oncogenic hindgut enterocytes in *Drosophila*

**DOI:** 10.1242/bio.061960

**Published:** 2025-06-17

**Authors:** Myrofora Panagi, Alexandros Galaras, Pantelis Hatzis, Yiorgos Apidianakis

**Affiliations:** ^1^ University of Cyprus, Department of Biological Sciences, 2109 Nicosia, Cyprus; ^2^ Institute for Fundamental Biomedical Research, Biomedical Sciences Research Center Alexander Fleming, 16672 Vari, Greece; ^3^ Department of Biochemistry and Biotechnology, University of Thessaly, 41500 Larissa, Greece

**Keywords:** EMT, Oncogene, Metastasis, Regenerative inflammation

## Abstract

Cancer cell invasion and subsequent metastasis account for most cancer related deaths. However, despite recent progress, there is a need to understand how the main pathways involved in oncogenic cell invasion and metastasis amalgamate into multifunctional networks. Using functional transcriptomic analysis of *Drosophila* Ras oncogenic hindgut enterocytes, we identify a feed-forward loop between the archetypical Toll/NF-κB pathway and Rac1 signalling driving actin cytoskeleton rearrangements, basement membrane degradation, and loss of intercellular adhesion. Our data support a signalling network in which Rac1, Toll and JNK signalling transmit the *Ras^V12^* signal that primes the hindgut enterocytes towards delamination and dissemination. Rac1 induces actin cytoskeleton signalling genes, *Rok*, *sqh*, *Apr2*, and *Apr3*, while JNK induces matrix metalloprotease-mediated basement membrane degradation and Toll induces *snail*-depended E-cadherin repression. Moreover, the Toll pathway positively regulates itself and the Rac1 pathway cytoskeletal genes downstream of the Ras oncogene, but JNK signalling alone does not suffice to induce cell dissemination. Notably, there is a tight crosstalk between Toll and Rac1 signalling that suffices to induce hindgut enterocyte invasiveness and has the key role in transmitting the *Ras^V12^* signal.

## Teaser

**Summary:** A feed-forward loop between Toll/NF-κB and Rac1 signalling drives Ras-induced cell invasion by coordinating cytoskeletal changes, adhesion loss, and basement membrane degradation in *Drosophila* hindgut enterocytes.

## INTRODUCTION

Immune responses play a dual role in tumour development: on the one hand, immune cells are recruited at the tumour site and induce apoptosis and elimination of cancer cells. On the other hand, the continuous release of pro-inflammatory factors establishes a microenvironment permissive to tumour growth. For example, constitutive NF-κB activity is reported in 40% of colorectal cancers and is associated with disease progression and poor outcomes (Lind et al., 2001; Sakamoto et al., 2009; Wu et al., 2015; Yu et al., 2004). NF-κB-mediated upregulation of chemoattractants at the pre-metastatic niche may influence the metastasis site by creating an environment that supports the seeding of tumour cells (Collins et al., 1995; Xia et al., 2014). Moreover, cytokines and growth factors produced by immune and epithelial cells alike elicit regenerative signalling that may feed intestinal dysplasia and tumorigenesis (Panayidou and Apidianakis, 2013; Kux and Pitsouli, 2014; Karin and Clevers, 2016). Chronic exposure to inflammatory responses enhances the accumulation of genetic mutations leading to genomic instability. As a result, cancer cells escape from immune-surveillance and acquire hallmarks of cancer cells (Hanahan and Weinberg, 2011). However, NF-κB signalling within pre-cancerous cells is pleiotropic, inducing cell proliferation, apoptosis, or metastasis depending on additional signalling pathways that are concomitantly activated.

*RAS* oncogene expression is the second most common genetic event in human colorectal cancers, found in more than a third of the metastatic stage cancers (Patelli et al., 2021). Constitutive activation of *RAS* promotes aberrant proliferation and growth, apoptosis, loss of differentiation, and invasiveness (Wan et al., 2019). However, this genetic pleiotropy points to context dependent modes of action of *RAS*, which may hinder anti-cancer drug specificity in the clinic, even if the *RAS* pathway is the drug target itself (Takashima and Faller, 2013). We have previously found that sustained infection facilitates the *Ras^V12^* signalling to induce basal invasion and dissemination of *Drosophila* hindgut cells to distant sites. Infection promotes dissemination by inducing part of the Imd innate immune pathway, upstream of Relish/NF-κB, which converges with *Ras^V12^* signalling on JNK pathway activation and concomitant extracellular matrix degradation (Bangi et al., 2012).

Taking advantage of a *Drosophila* hindgut cell invasion and dissemination model, we pinpoint transcriptional, post-transcriptional, and cellular changes which explain the transition of *Ras^V12^* oncogene-expressing hindgut enterocytes regardless of infection from the normal-healthy to an abnormal-invasive state through a crosstalk between the archetypical Toll/NF-κB and Rac1 signalling.

## RESULTS

### *Ras^V12^* expression induces cytological and morphological alterations in the hindgut

**Fig. 1.**
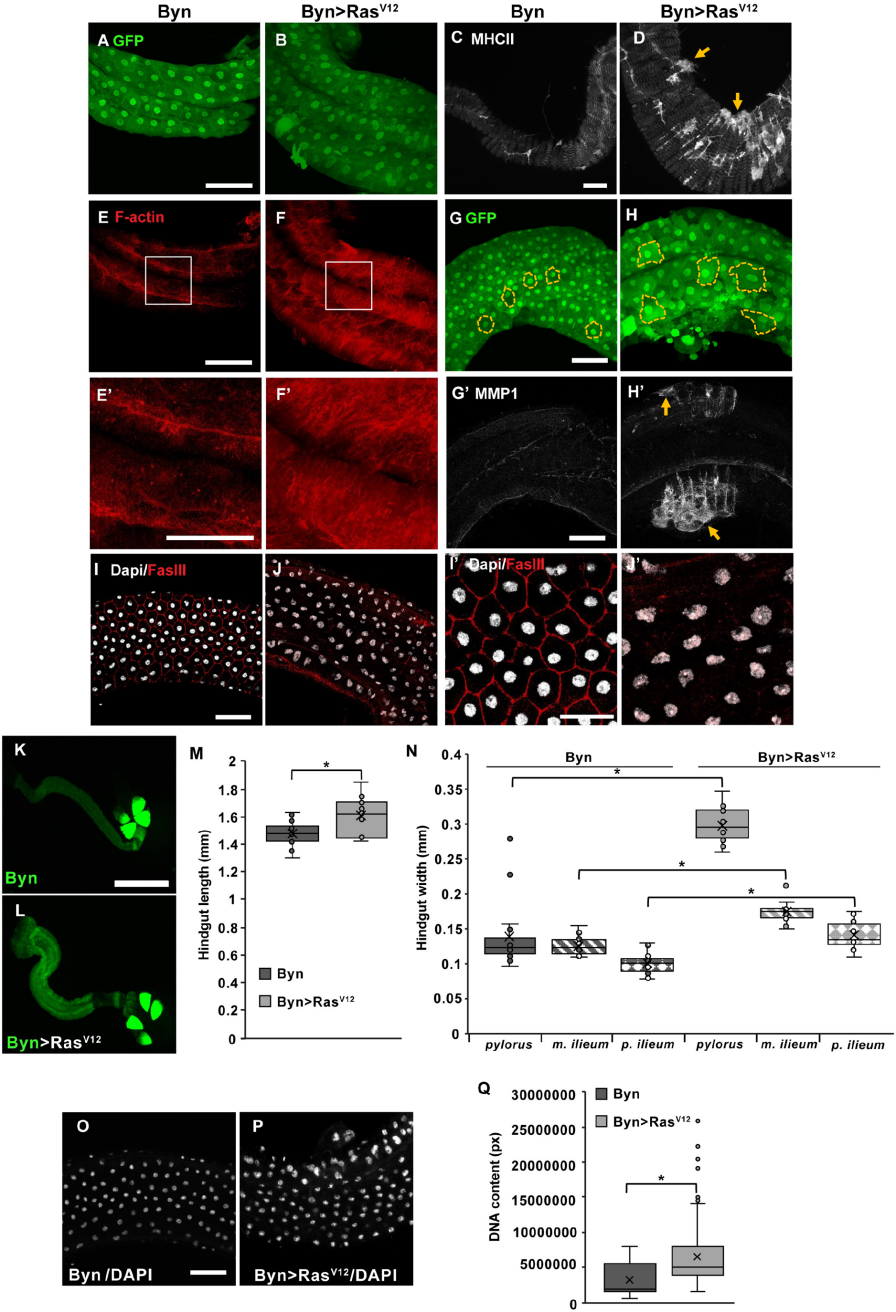
**Cytological and morphological alterations induced by *Ras^V12^* expression in hindgut enterocytes.** (A-I′) Fluorescently marked wild-type control (Byn) and oncogenic (Byn>Ras^V12^) hindguts. (A,B,E-F′) *Ras^V12^* versus control GFP-expressing hindgut enterocytes (A,B) exhibit denser F-actin viewed via the lifeact-Ruby reporter under the same settings (E,F). E′ and F′ correspond to the magnified frames shown in E and F. (C,D) MHCII expression with arrows indicating foci of high MHC II expression in the delamination areas. (G,H,G′H′) *Ras^V12^* expression changes the size and shape of hindgut enterocytes (marked with yellow dashed lines in G,H). MMP1 expression with arrows indicating foci of high MMP1 expression in the delamination areas. (I,J,I′J′) FasIII immunostaining indicating widespread loss of FasIII from *Ras^V12^* enterocytes at standard (I,J) and higher (I′,J′) magnification. FasIII loss is not localized to the delamination areas. (K,L) *Ras^V12^*- and GFP-expressing and control hindguts. (M,N) *Ras^V12^* hindguts are longer and wider (*n*=15-20). (O,P) Representative fluorescence images of DAPI staining at the middle hindgut region. O is the same image as I, showing only DAPI staining. (Q) Nuclear DNA content of *Ras^V12^* expressing versus control hindgut enterocytes (*n*=195-215 nuclei/genotype). Scale bars: 50 μm, error bars: s.d., Student’s *t*-test and one-way ANOVA: *P<0.05.

To assess the cytological priming of hindgut enterocytes prior to their delamination upon *Ras^V12^* oncogene expression, we used two markers: (i) F-actin reporter in *w;UAS-Ras^V12^/+;byn-Gal4,tub-Gal80^ts^,UAS-gfp/UAS-lifeact-Ruby* and control flies, and (ii) Fasciclin III (FasIII), a septate junction protein via antibody staining of *w;UAS-Ras^V12^/+;byn-Gal4,tub-Gal80^ts^,UAS-gfp/+* and control flies (Fig. 1D-D′). We find a higher density of F-actin (Fig. 1E,E′,F,F′), and a widespread loss of FasIII in response to *Ras^V12^* expression (Fig. 1I,I′G,G′).

To assess the invasive characteristics of the oncogenic hindgut enterocytes at the delamination foci, we stained for non-muscle myosin II heavy chain (MHC-II) (Fig. 1C,D), and matrix metalloproteinase 1 (Mmp1) (Fig. 1G,G′,H,H′). We find high MHC II and MMP1 expression in the delamination areas as indicated with arrows in Fig. 1D and H′, respectively.

Noticeably, *Ras^V12^* expressing hindguts are wider and longer than wild type (Fig. 1K-N), which can be partly attributed to increased cell growth via endoreplication, indicated by larger nuclei and higher DNA content per nucleus (Fig. 1O-Q). This is corroborated by the biggest and irregularly shaped enterocytes bearing bigger nuclei, as shown in Fig. 1A,B,G,H,I-J′,O,P. To emphasize, we circumscribe cell borders in Fig. 1G,H with dotted lines. To assess whether the increased width of the ileum is accompanied by more cells, we quantified the number of cells per ileum length and find that *Ras^V12^* expressing hindguts exhibit tentatively higher, that is, 91.3±10.7 s.d. (*n*=7) versus 81.8±23.6 s.d. (*n*=5) of the control per 200 μm of middle ileum length. However, the difference is not significant (*P*=0.52, Mann–Whitney *U*-test). We conclude that the bigger dimensions of the hindgut ileum upon *Ras^V12^* expression are primarily due to enterocyte growth.

### Differential expression of cytoskeleton, innate immunity and stress, and cell polarity and adhesion genes upon *Ras^V12^* expression in the hindgut enterocytes

**Fig. 2.**
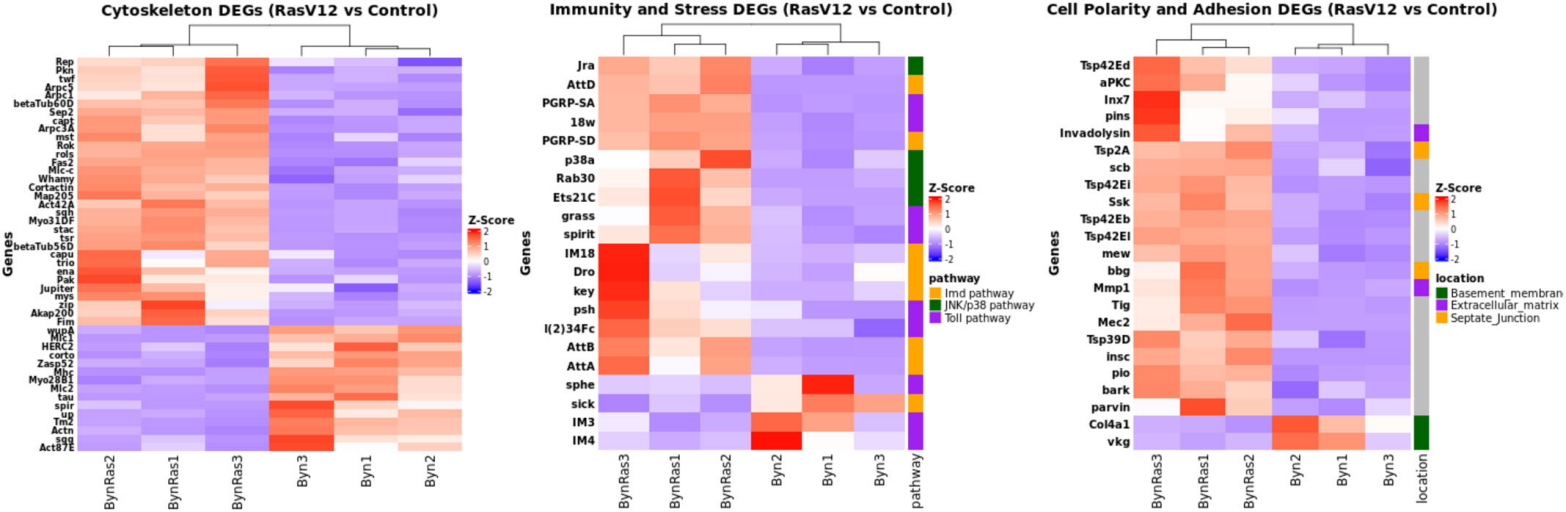
**Heatmaps of DEGs belonging in the cytoskeleton, immunity and stress, and cell polarity and adhesion functional categories.** All three replicates per condition and genes by conditions-replicates clustering are shown. Cytoskeleton genes are upregulated in *byn-Gal4 UAS-Ras^V12^* hindguts, but those typical of skeletal muscle cells are downregulated. Immunity and stress genes of the Toll, Imd and JNK/p38 pathways are predominantly upregulated by *Ras^V12^*. Cell polarity and adhesion genes related to septate junctions and extracellular matrix are upregulated, while the basement membrane genes *Col4a1* and *vkg* are downregulated.

**Fig. 3.**
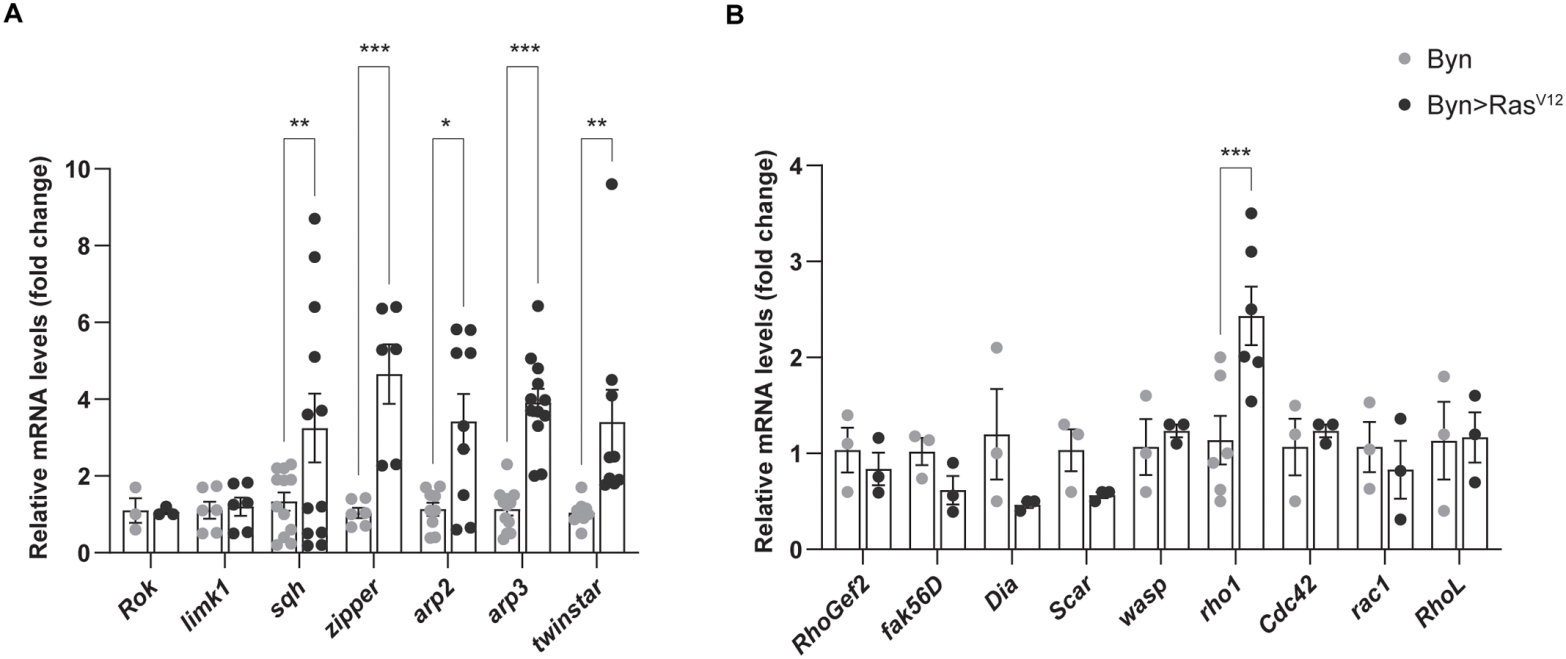
**Rho pathway gene induction in control versus *Ras^V12^* flies via RT-qPCR.** (A) Validation of Rho signalling genes assigned to the ‘cytoskeleton’ cluster. (B) Screening for additional differentially expressed genes of the Rho signalling pathway in control versus *Ras^V12^* flies. Student’s *t*-test: *P<0.05, **P<0.01, ***P<0.001.

To explore the signalling network involved in the transformation of *Ras^V12^* oncogene-expressing hindgut enterocytes we performed RNA-Seq. Comparison between hindguts expressing *Ras^V12^* in their enterocytes (*w;UAS-Ras^V12^/+;byn-Gal4,tub-Gal80^ts^,UAS-gfp/+*) and control hindguts (*w;;byn-Gal4,tub-Gal80^ts^,UAS-gfp/+*) reveals differentially expressed genes (DEGs) at a *P*-value threshold of 0.05, all off which, but three, exhibited a log2 fold change >1 or <−1. Functional annotation reveals 47 deregulated genes within a ‘cytoskeleton’ functional cluster, many of which are small Rho-GTPase (Rho, Rac, Cdc42) signalling genes, including *twinstar* (*tsr*), *actin-related protein 2/3 complex subunit 1*, *actin-related protein 2/3 complex subunit 3A* and *actin-related protein 2/3 complex subunit 5*, *Rho kinase* (*rok*), non-muscle myosin II heavy chain *zipper* (*zip*), and non-muscle myosin II regulatory light chain *spaghetti squash* (*sqh*), as well as FasII and Cortactin (Table S1 and Fig. 2). Tsr, the cofilin homologue, promotes F-actin turnover and actin depolymerization from the pointed end, Rok is the effector kinase of Rho1, while the Arp2/3 complex is necessary for branched actin nucleation and formation of filopodia and lamellipodia. The relative increase in mRNA levels of *sqh*, *zip*, *Arp2*, *Arp3*, *tsr*, and *Rho1* is further confirmed in RT-qPCR experiments (Fig. 3A,B). Innate immunity and stress DEGs include: (i) Toll signalling pathway and target genes, *Gram-positive Specific Serine protease* (*grass*), *spirit*, *spheroide* (*sphe*), *Peptidoglycan Recognition Protein SA* (*PGRP-SA*), *18 wheeler/Toll-2* (*18w*), *Persephone* (*psh*), *l(2)34Fc, IM3* (*BomS3*), *IM4* (*Dso1*) and *Baramicin A1* and *A2* (*BaraA1, BaraA2*), (ii) immune deficiency (Imd) pathway and target genes, *Peptidoglycan Recognition Protein SD* (*PGRP-SD*), *kenny* (*key*), *Attacins A*, *B*, *D* (*AttA*, *AttB*, *AttD*), *Drosocin* (*Dro*) and *Neuron navigator* (*Nav* or *sick*), (iii) JNK pathway and target genes, *Jun-related antigen (Jra*), *Ets at 21C* (*Ets21C*), and *Rab30* (Table S2 and Fig. 2). Cell polarity and adhesion DEGS include: the collagen IV basement membrane genes, *Cg25C* (*Col4a1*) and *viking* (*vkg*), the septate junction genes, *Tetraspanin2A* (*Tsp2A*), *big bang* (*bbg*), *mesh*, *Snakeskin* (*Ssk*) and *lethal (2) giant larvae* (*l(2)gl*), and the matrix metalloproteinases*, Mmp1* and *Invadolysin* (Table S3 and Fig. 2).

### *Ras^V12^* expression induces enterocyte dissemination via small Rho-GTPase signalling

**Fig. 4.**
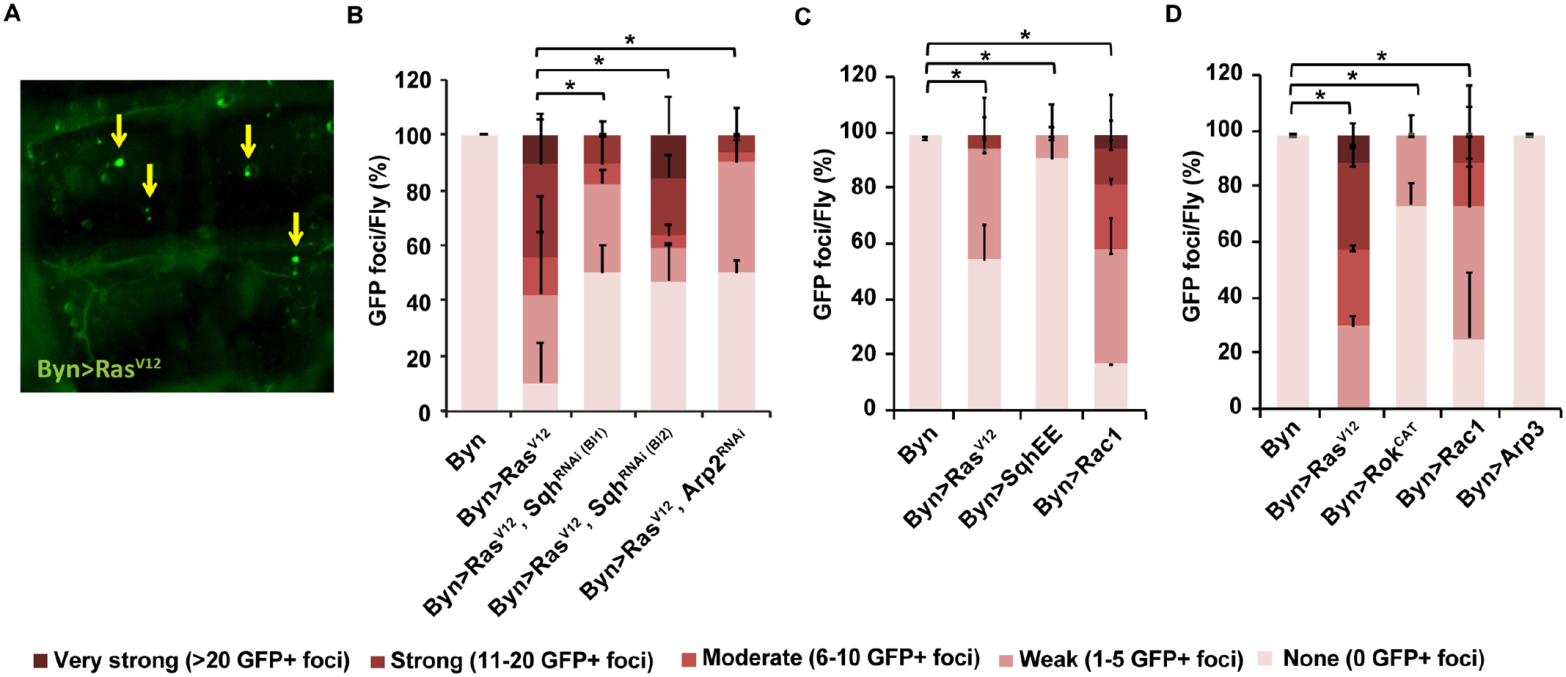
**Rac1 signalling is necessary and sufficient for *Ras^V12^* hindgut enterocyte dissemination.** (A) Foci indicated by arrows of *Ras^V12^*- and GFP-expressing hindgut enterocytes disseminated in the fly abdominal cavity, 14 days post transgene induction at 29°C. Arrowhead length: 40 μm. (B-D) Foci quantification via direct tissue inspection under the fluorescent stereoscope. (B) Downregulation of small Rho-GTPase signalling responses reduces dissemination induced by *Ras^V12^*. (C,D) Constitutively active small Rho-GTPase signalling for 7 days (C) and 14 days (D) suffices to induce dissemination. *n*=6 replicates, 15 flies per replicate; error bars: s.d. Fisher's exact test with a 5×2 contingency table: *P<0.05.

To assess their involvement in hindgut enterocyte fate upon *Ras^V12^* expression, we downregulated via RNAi the small Rho-GTPases, *sqh* and *Arp2* for 14 days. In all cases, RNAi caused a significant reduction in hindgut enterocyte dissemination (Fig. 4A,B). Moreover, activated *Rac1* (*Rac1^V12^*) alone sufficed to induce strong cell dissemination at 7 days (Fig. 4B) and at 14 days (Fig. 4C), while activated *Rok* (*Rok^CAT^*) expression induced mild cell dissemination at 14 days (Fig. 4C). We conclude that *Rac1* is necessary and sufficient for cell dissemination, while genes described to act downstream, *sqh* and *arp2*, may facilitate Rac1 action.

**Fig. 5.**
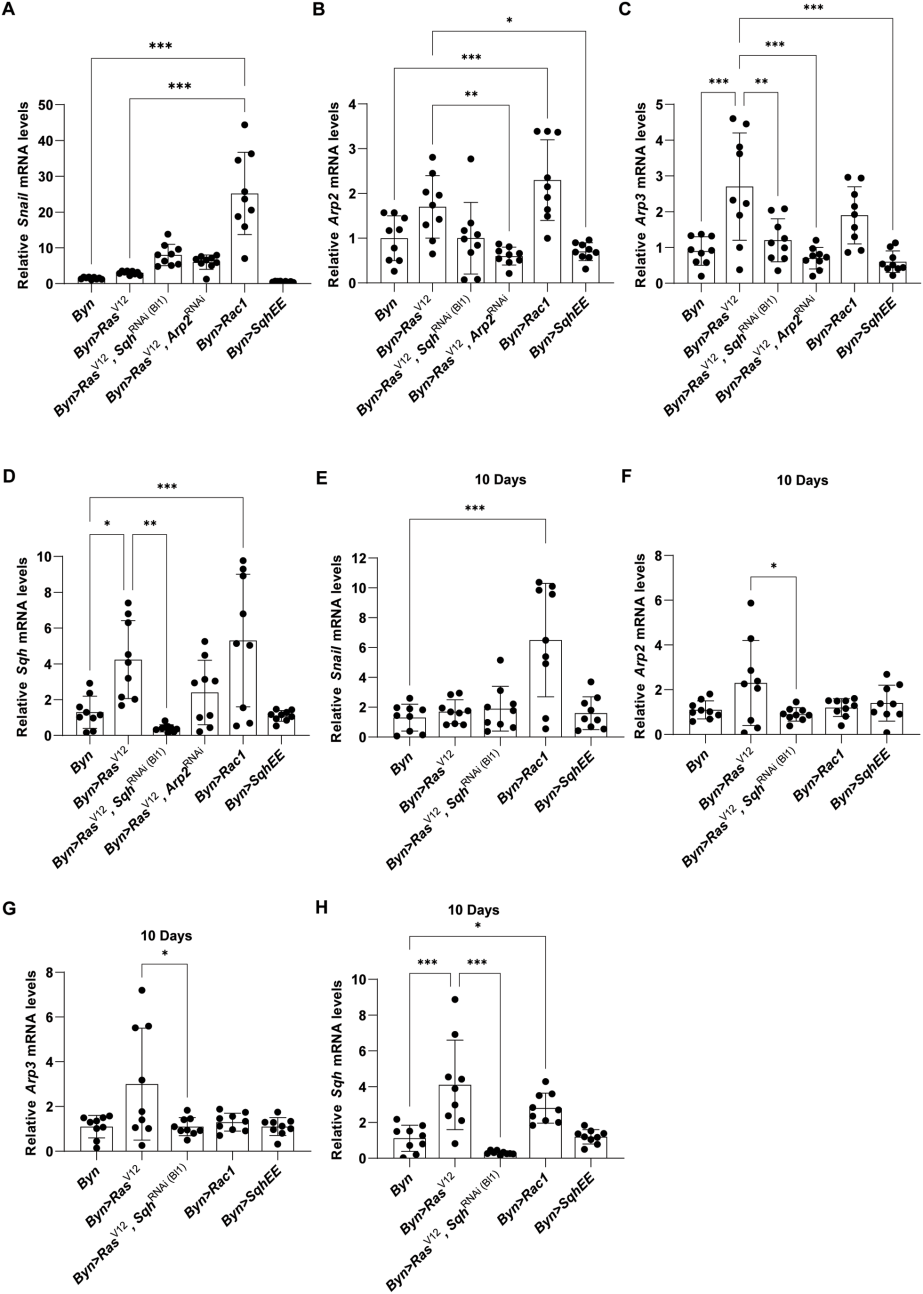
***Ras^V12^* induces *snail*, *Apr2*, *Apr3* and *sqh*, but also the regulation of *Arp2* and *Arp3* by *sqh* and *Arp2*.** (A,E) Rac1 facilitates cell migration via the regulation of *snail* expression at 10 (E) and 14 (A) days of induction. (B,C) *Arp2* and *Arp3* expression is regulated by both Rac1 and the Rho signalling cascade 14 days post induction. (F,G) *Arp2* and *Arp3* expression remains relatively unaffected at 10 days of oncogene induction. (D,H) *sqh* expression is regulated by Rac1 and Rho-GTPase signalling at 10 (D) and 14 (A) days. *n*=3 biological replicates, 100 hindguts per replicate; error bars: s.d. One-way ANOVA: *P<0.05, **P<0.01, ***P<0.001.

Considering potential targets of Rac1 signalling, we assessed the expression of *snail*, a master regulator of epithelial to mesenchymal transition (EMT) implicated in tumour aggressiveness. We noticed a prominent increase in *snail* expression when Rac1 activity was induced for 10 and 14 days (Fig. 5A,E). Moreover, Rac1 was sufficient to induce *Arp2*, *Arp3* and *sqh* (Fig. 5D,H), while *Arp2* and *Arp3* induction by *Ras^V12^* required *sqh* and *Arp2* (Fig. 5B,C,F,G), pointing to a positive regulation among *Arp2*, *Arp3* and *sqh*. However, the *Arp2* and *sqh* genes were not required for *snail* induction in *Ras^V12^* enterocytes (Fig. 5A,E). Thus, *Ras^V12^* induces *snail*, *Apr2*, *Apr3* and *sqh*, but also the regulation of *Arp2* and *Arp3* by *sqh* and *Arp2*.

### Toll signalling induces and facilitates *Ras^V12^* promoted enterocyte dissemination

**Fig. 6.**
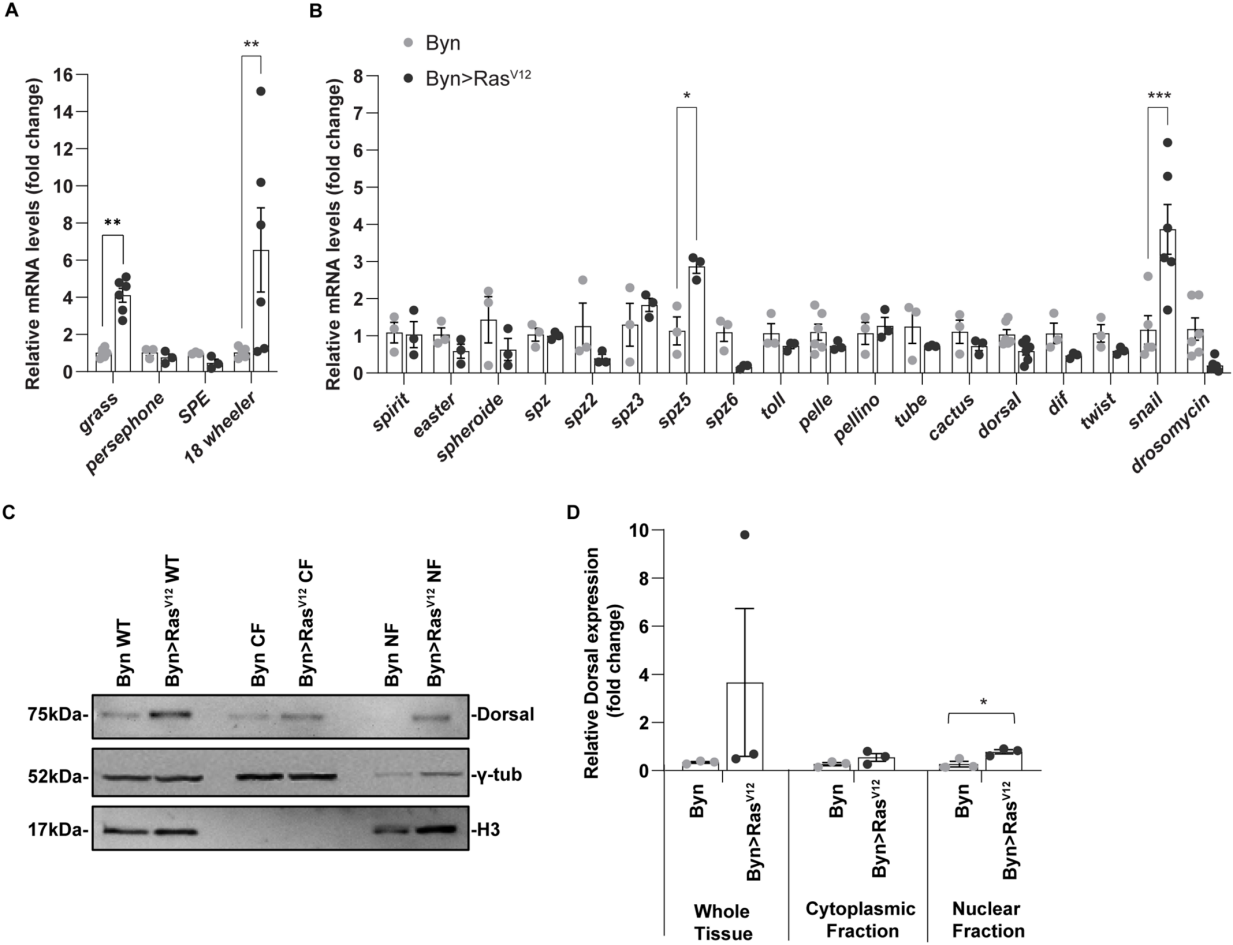
**Induction of Toll pathway genes, *grass*, *spz5* and *snail*, and nuclear translocation of dorsal by *Ras^V12^*.** (A,B) Validation of Toll signalling genes assigned to the ‘Innate immunity and stress’ cluster (A) and screening for additional differentially expressed Toll signalling genes in control and *Ras^V12^* hindguts via RT-qPCR. (C) Western blot analysis of dorsal cytoplasm/nucleus fractionation in *byn-Gal4* and *byn-Gal4*/*UAS-Ras^V12^* flies induced for 14 days. γ-tub, gamma tubulin and H3, histone 3 are loading controls for cytoplasmic (CF) and nuclear fractions (NF), respectively. Cell lysates were prepared from 100 hindguts per genotype. (D) Quantification of protein expression in whole tissue (WT) extract, CF and NF from biological triplicates via ImageJ software. Student’s *t*-test: *P<0.05, **P<0.01, ***P<0.001.

To validate the induction of Toll/Toll-like pathway genes by *Ras^V12^* (Table S2), we assessed by RT-qPCR the activation of these genes in hindguts expressing *Ras^V12^* in their enterocytes. We observed significant induction of the expression of *18w*, *grass*, *spz5* and *snail*, pointing to their contribution in oncogenic cell formation (Fig. 6A,B). In line with these results, cytoplasm to nucleus fractionation experiments show that *Ras^V12^* expression increases the protein level of nuclear Dorsal/NFκ-B, a transcription factor integral to Toll signalling (Fig. 6C,D). While upstream and downstream genes, *spz5* and *snail*, respectively, are transcriptionally induced by *Ras^V12^*, the lack of induction of *spz*, *Toll*, *tube*, *pelle*, *dif* and *dorsal* Toll at the mRNA level (Fig. 6B) points to a post-transcriptional activation of the core Toll pathway genes.

**Fig. 7.**
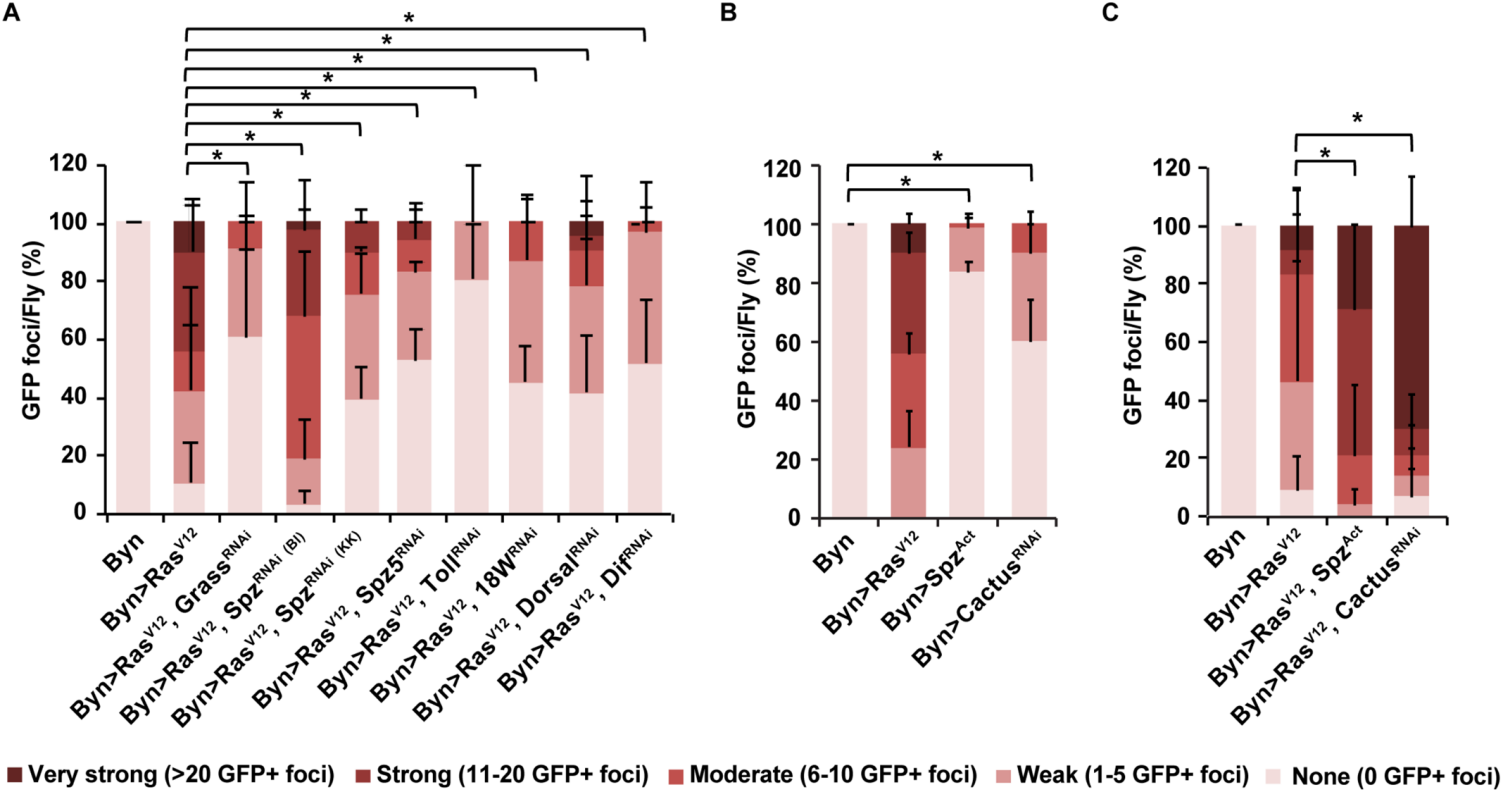
**Toll signalling is necessary and sufficient for *Ras^V12^* hindgut enterocyte dissemination.** (A-C) Quantification of GFP-expressing hindgut enterocytes disseminated in the abdominal cavity of flies 14 days *Ras^V12^* induction. Dissemination phenotypes are assigned into one of the following categories based on the number of foci counted (*n*=3 replicates, 15 flies per replicate; error bars: s.d.). (A) Dissemination induced by *Ras^V12^* is suppressed upon Toll signalling downregulation. (B) Constitutively active Toll signalling suffices to induce cell delamination. (C) Synergistic effects of Toll and *Ras^V12^*. *P*-values were calculated using the Fisher's exact test with a 5×2 contingency table to assess the five phenotypic categories: *P<0.05.

Next, we examined the necessity and sufficiency of signalling through the Toll pathway for hindgut enterocyte dissemination, with and without *Ras^V12^* oncogene expression. We find that silencing the extracellular Toll pathway components *grass*, *spz*, and *spz5*, the receptors *Toll* and *18w*, and the Toll pathway NF-κΒ transcription factors *Dif* and *dorsal*, causes a decrease in cell dissemination. The decrease is dramatic upon expression of Toll^RNAi^, highlighting the involvement of Toll innate immune signalling in the process (Fig. 7A). Accordingly, constitutive activation of Toll signalling by expressing an activated form of *spz* (*spz^Act^*) or *cactus* downregulation via RNAi (*cactus^RNAi^*) induces low but significant cell dissemination even in the absence of *Ras^V12^* oncogenic signalling (Fig. 7B). Moreover, co-expression of *Ras^V12^* with *spz^Act^* or *cactus^RNAi^* enhances oncogenic cell dissemination (Fig. 7C).

### Crosstalk between Toll signalling and small Rho-GTPases in the hindgut enterocytes

**Fig. 8.**
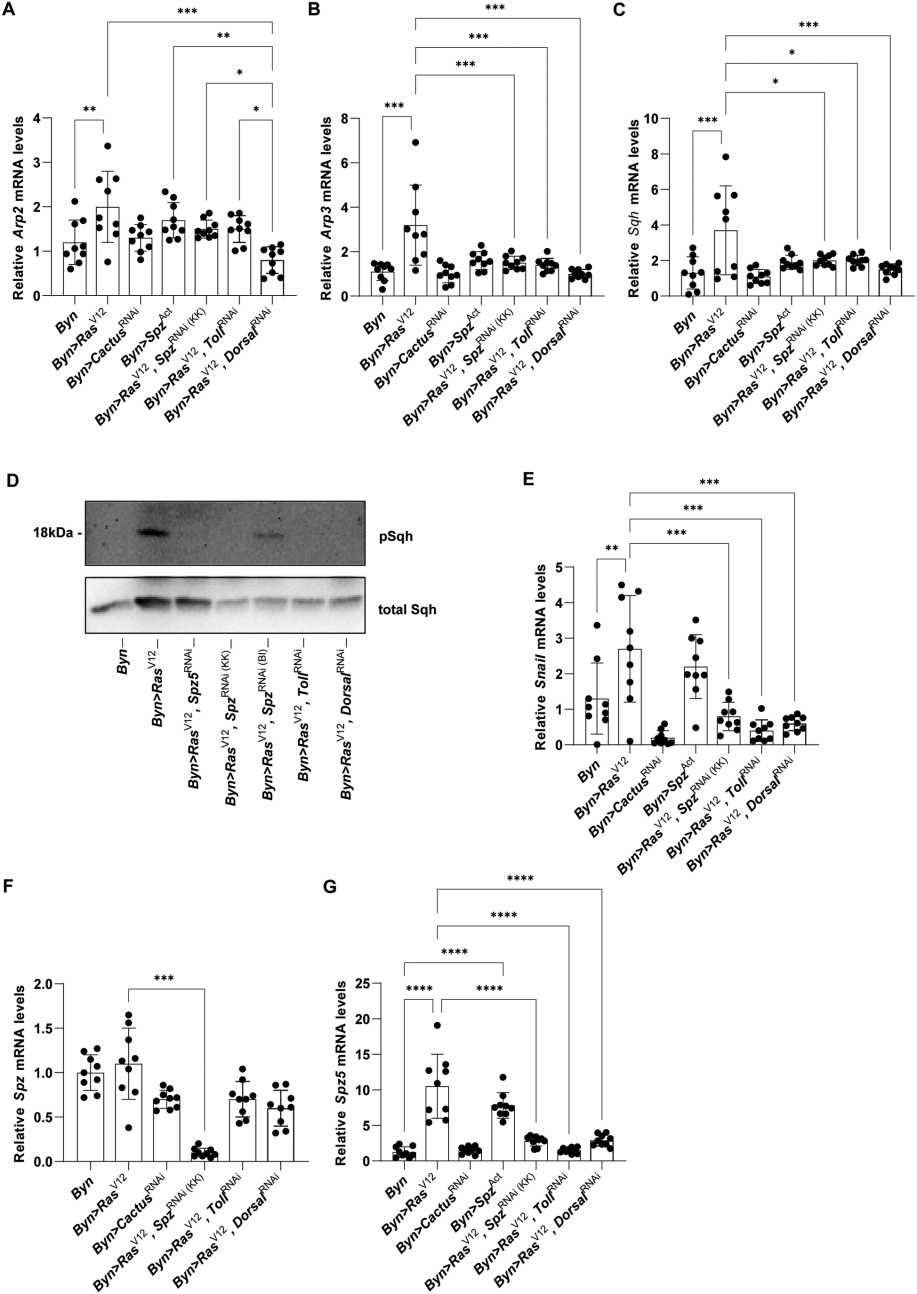
**Toll signalling induces itself and mediates cytoskeleton gene induction by *Ras^V12^*.** Expression of *Arp2* (A), *Arp3* (B) and *sqh* (C) upon transgene induction via RT-qPCR. (D) Representative immunoblotting against p-Sqh (*n*=3). Total Sqh was used as a loading control. Quantification of Toll transcriptional targets *snail* (E), *spz* (F) and *spz5* (G). *n*=3 biological replicates, 100 hindguts per replicate; error bars: s.d., one-way ANOVA: *P<0.05, **P<0.01, ***P<0.001, ****P<0.0001.

To investigate whether there is a crosstalk between Toll and the small Rho-GTPase responses, we genetically modified the Toll pathway and assessed *Arp2*, *Arp3* and *sqh* expression*.* We find that the expression of the three cytoskeletal genes in the hindgut is significantly reduced upon *spz*, *Toll* or *dorsal* downregulation in oncogenic hindgut enterocytes, while *Arp2* can be induced by constitutively activated *spz* (*UAS-spz^Act^*) alone (Fig. 8A,B,C). Moreover, (i) *spz*, *spz5*, *Toll* or *dorsal* downregulation impedes Sqh phosphorylation induced by *Ras^V12^* (Fig. 8D), (ii) *spz*, *Toll* and *dorsal* downregulation reduces *snail* expression induced by *Ras^V12^*, and (iii) *spz^Act^* alone can induce *snail*, pointing to a mechanism through which Toll signalling promotes oncogenic cell dissemination (Fig. 8E). Interestingly, although *spz* expression does not increase upon *Ras^V12^* expression (Fig. 8F), *spz5* expression increases upon either *Ras^V12^* or *spz^Act^* expression, and decreases to baseline upon *spz*, *Toll* or *dorsal* downregulation in *Ras^V12^* expressing flies (Fig. 8G). Spz5 was first identified as the ligand of Toll-6, but it also binds and activates Toll (Mishra-Gorur et al., 2019). Thus, Toll signalling regulates itself, *snail* and cytoskeletal genes downstream of Rac1 in hindgut enterocytes.

**Fig. 9.**
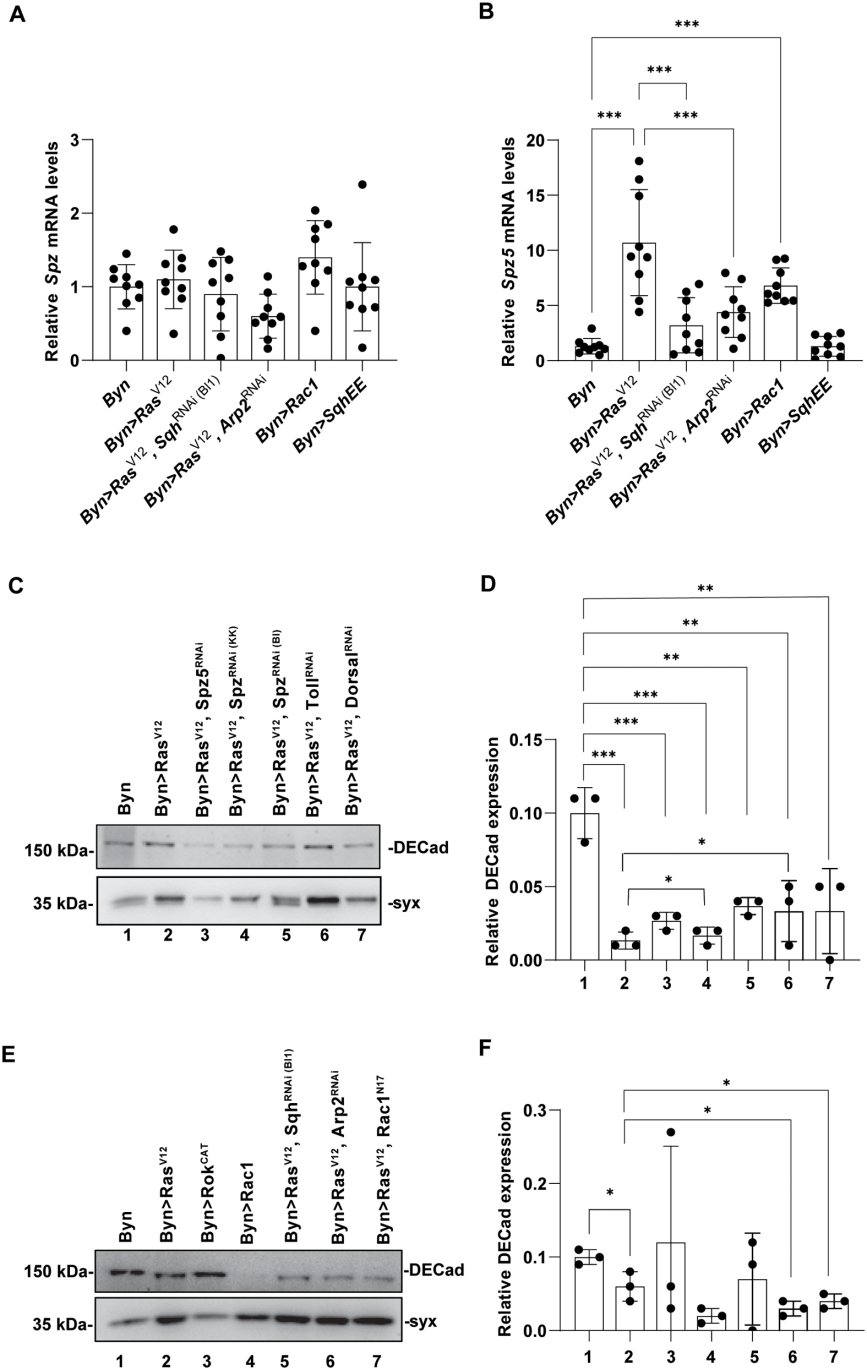
***Ras^V12^* and Rac1 induce Toll signalling and *Ras^V12^* signals through Toll to repress DE-cadherin.** Expression of *spz* (A) and *spz5* (B) upon transgene induction via RT-qPCR. (C,D) Immunoblotting with antibodies against E-cadherin and quantification of expression following downregulation of Toll signalling. (E,F) Immunoblotting with antibodies against E-cadherin and quantification of expression following downregulation of small Rho-GTPase responses. *n*=3 biological replicates, 100 hindguts per replicate; error bars: s.d., one-way ANOVA: *P<0.05, **P<0.01, ***P<0.001.

To test whether Rac1 signalling genes can affect the Toll pathway, we measured the expression of *spz* and *spz5* upon cytoskeletal gene downregulation and induction. Although s*pz* expression did not change in *Ras^V12^* expressing hindguts upon cytoskeletal gene downregulation or upon *sqh* induction (Fig. 9A), *spz5* expression was decreased by 3-fold in *Ras^V12^* expressing hindguts upon *sqh^RNAi^* or *Arp2^RNAi^* expression (Fig. 9B). Nonetheless, both *spz* and *spz5* contributed to the repression of DE-cadherin (Fig. 9C,D). DE-cadherin protein levels are strikingly reduced in *Ras^V12^* expressing hindguts, while downregulation of any of the two ligands, *spz* or *spz5*, results in significant de-repression of DE-cadherin (Fig. 9C,D). However, while *Rac1* downregulation tentatively represses DE cadherin, similarly to *Ras^V12^*, inhibition via *sqh^RNAi^* or *Arp2^RNAi^* in the presence of *Ras^V12^* does not derepress DE-cadherin (Fig. 9E,F), suggesting that Toll signalling is repressing DE-cadherin directly.

### Toll and Rac1 signalling promote hindgut cell dissemination via JNK signalling activation

**Fig. 10.**
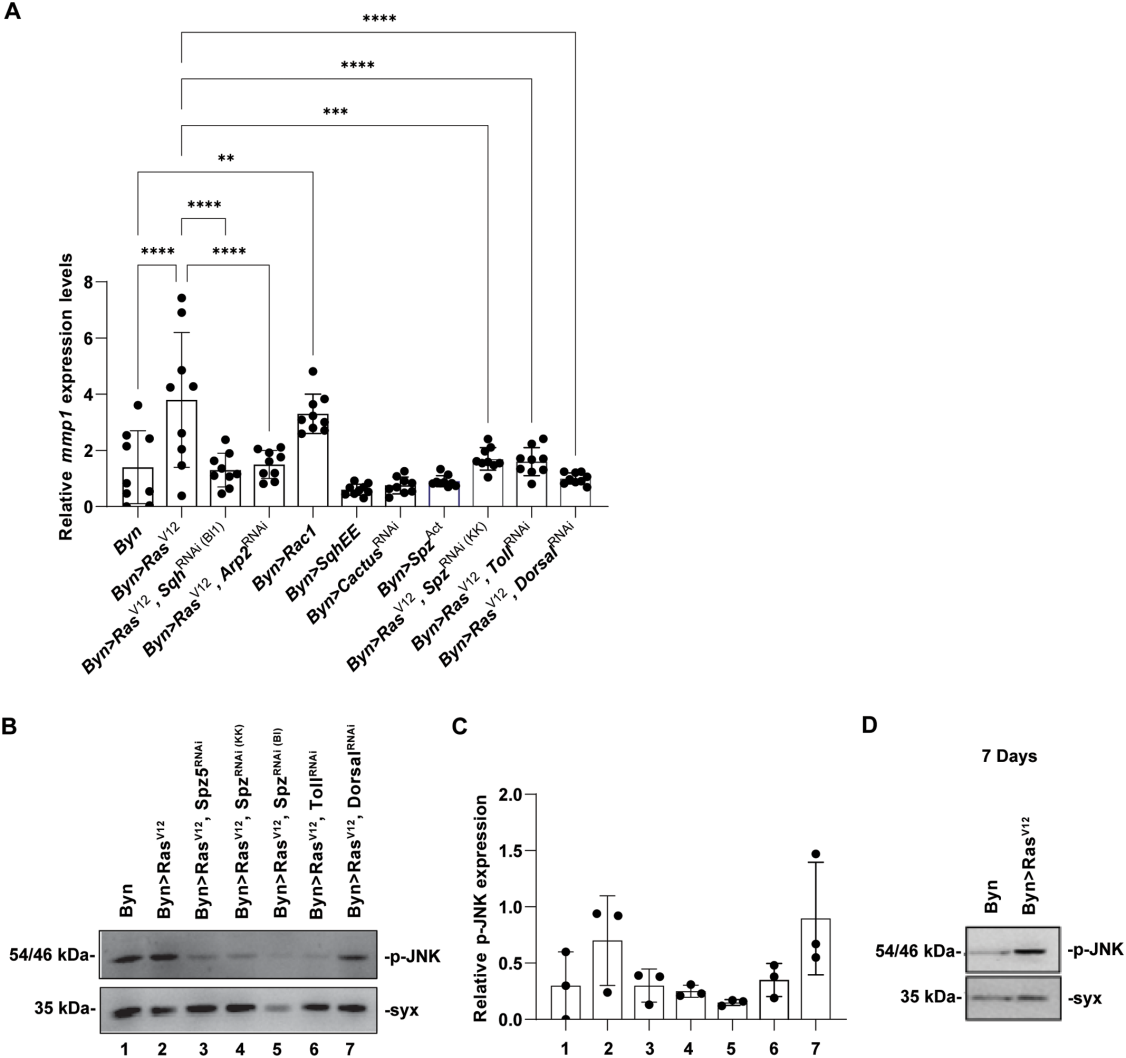
**Rac1 and Toll signalling induce activated JNK and Mmp1.** (A) Expression of the JNK transcriptional target *Mmp1* upon transgene induction via RT-qPCR. (B,C) Representative immunoblotting and quantification of p-JNK protein after silencing of Toll immune signalling in the presence of *Ras^V12^* at 14 days induction. (D) Western blotting against p-JNK after 7 days of *Ras^V12^* induction, shows increased JNK phosphorylation in *Ras^V12^* expressing flies. Syx, Syntaxin as a loading control. *n*=3 biological replicates, 100 hindguts per replicate; error bars: s.d., one-way ANOVA: *P<0.05, **P<0.01, ***P<0.001, ****P<0.0001.

Invasion of primary tumour cells in neighbouring tissues requires the degradation of basement membrane. Accordingly, MMP1 protein is induced in delaminating *Ras^V12^* hindgut cells (Fig. 1G,H), and *Mmp1* expression is higher in *Ras^V12^* hindguts (Fig. 10A). Interestingly, downregulation of *sqh* or *Arp2* in *Ras^V12^* enterocytes reduces *Mmp1* expression to baseline levels, whereas activated Rac1 alone mimics the effect of *Ras^V12^*. In line with our hypothesis that Rac1 and Toll signalling crosstalk, downregulation of *spz*, *Toll* and *dorsal* inhibits the induction of *Mmp1* by *Ras^V12^* (Fig. 10A).

To validate the role of the Toll pathway in JNK activation, a positive regulator of Mmp1 (Bangi et al., 2012), we performed western blotting with antibodies against phosphorylated JNK (p-JNK). We find an increase in the levels of p-JNK upon *Ras^V12^* expression and a reduction of this activation upon downregulation of *spz*, *spz5* and *Toll*, but not *dorsal*, suggesting that JNK activation by *Ras^V12^* requires upstream Toll signalling (Fig. 10B-D). Notably, we normalized p-JNK levels to those of the ‘housekeeping’ protein Syntaxin to indicate the relative amount of phosphorylated JNK within the cells, attributable to the total amount of JNK protein and the fraction of it being phosphorylated.

**Fig. 11.**
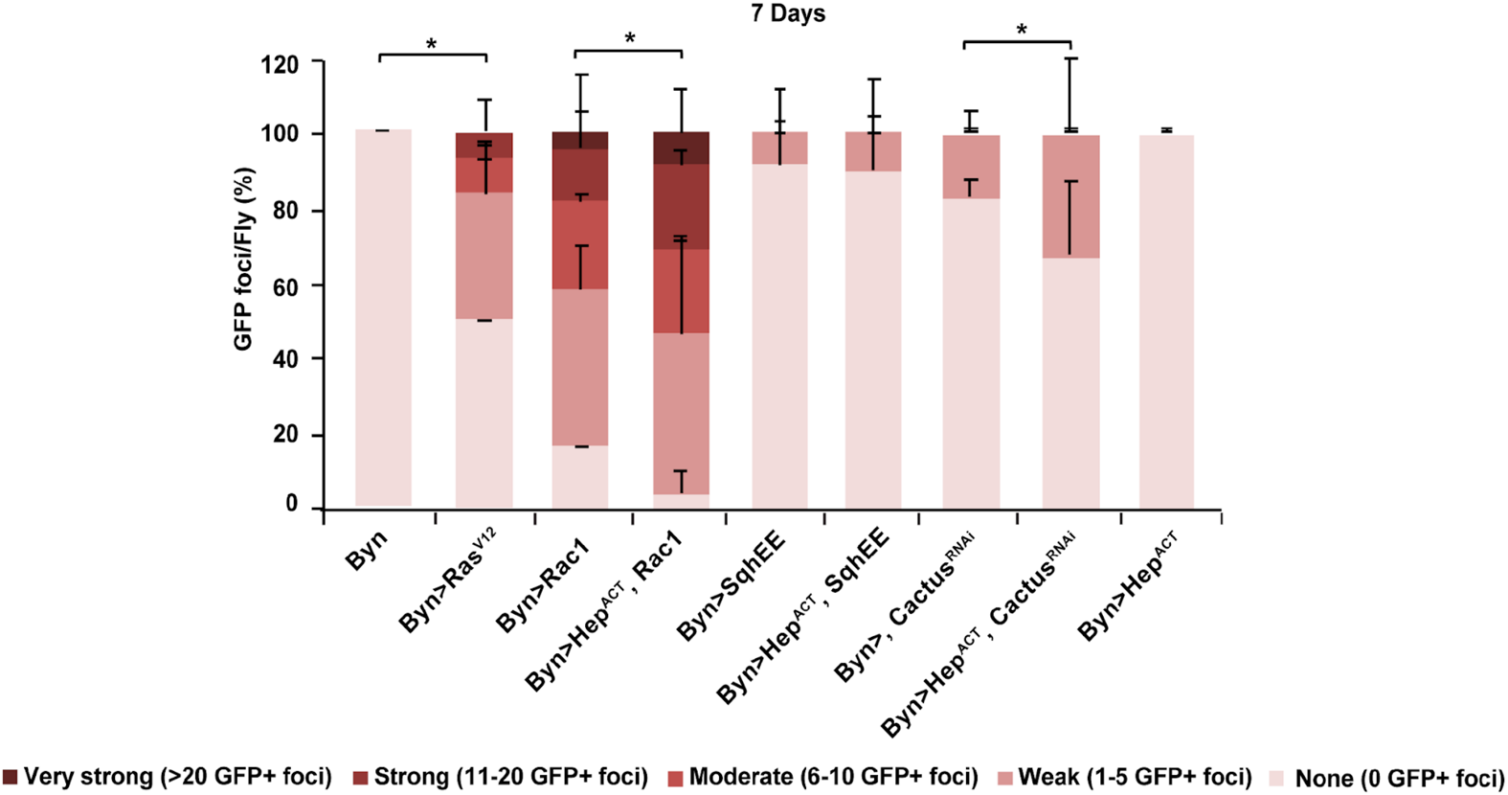
**JNK signalling facilitates is necessary but not sufficient for Toll/Rac1-induced hindgut enterocyte dissemination.** Quantification of GFP-expressing hindgut enterocytes disseminated in the abdominal cavity of flies at 7 days of Toll or Rac1 induction. *n*=3 replicates, 15 flies per replicate; error bars: s.d. Fisher's exact test with a 5×2 contingency table: *P<0.05.

To assess whether JNK facilitates cell dissemination mediated by Toll or Rac1, we induced Toll signalling via *cactus^RNAi^* and Rac1 signalling via *Rac1^V12^* or *sqhEE*, in combination with JNK signalling activation via *hep^Act^* in hindgut enterocytes and assessed cell dissemination at 7 days of transgene induction (Fig. 11). No delamination was observed by JNK pathway activation alone, and no synergy was found by simultaneous induction of *sph*. However, JNK activation enhanced the effect of Rac1 or Toll signalling on dissemination (Fig. 11). This agrees with previous findings showing that JNK activation enhances *Ras^V12^* induced dissemination (Bangi et al., 2012). Thus, JNK activity facilitates but does not suffice for cell dissemination.

## DISCUSSION

Constitutively active mutant forms of Ras are found in more than a third of human cancers and in as many metastatic colorectal cancers (Murugan et al., 2019; Patelli et al., 2021). Thus, targeting Ras signalling is an attractive therapeutic strategy. However, pharmacological targeting of Ras signalling is hampered by the alternative ways it is activated (Samatar and Poulikakos, 2014). Moreover, constitutively active Ras tumours induce cell death in surrounding cells, promoting Ras clonal expansion (Moreno et al., 2019). A more sophisticated targeting of Ras signalling may be achieved through combinatorial strategies that are directed at multiple nodes of the signalling network.

Here, we used the *Drosophila* hindgut epithelium model to reveal adjunct nodes of the pro-metastatic *Ras^V12^* signalling as additional targets of therapeutic intervention. *Drosophila Ras^V12^* triggers cell entry into the S phase and promotes tissue overgrowth and tissue invasion, recapitulating properties of mammalian cancer development (Prober and Edgar, 2002; Bangi et al., 2016, 2012). Our transcriptomic analysis was performed on day 7 post *Ras^V12^* induction, when enterocyte dissemination is still low per previous findings (Bangi et al., 2012), and suggested genes in at least three functional categories: (1) cytoskeleton, (2) immunity and stress and (3) cell polarity and adhesion as mediators of *Ras* oncogene signalling on cell motility. These were statistically significantly altered in expression (most of them orders of magnitude below the *P*<0.05 limit) and increased between 2.2- and 6.9-fold, 2.3- and 273-fold, and 2.1- and 27-fold (Tables S1-S3), respectively. However, the signalling pathways uncovered by this, and further analysis involve genes that are not necessarily activated by *Ras^V12^* at the transcription level. Many receptors and kinases are generally not at all activated transcriptionally. For example, the transcription factor of the Toll pathway, Dorsal, is activated via nuclear translocation. Thus, transcriptional activation for many of the genes studied is suggestive rather than a prerequisite for their involvement in the process of tissue transformation.

Previous findings indicated that the *Ras* oncogene signalling effect on cell motility is mediated by Rac1 signalling and downstream cytoskeletal rearrangements via the polymerization of actin branches and cellular contraction (Ridley, 2001; Brumby et al., 2011). To validate the role of Rac1 signalling in our model, we performed experiments showing that Rac1 is necessary and sufficient for cell dissemination. Rac1 acts as a master regulator of all aspects of cell dissemination downstream of *Ras^V12^* activation via a *Rac1-Rho-Rok-sqh/zipper/tsr/Arp2/3* pathway, which orchestrates actin and stress fibre formation, consistent with previous studies on *Drosophila* hemocytes and development (Williams et al., 2007; Verdier et al., 2006).

Of note, *Ras^V12^* and *Ras^V12^ Apc^null^ Drosophila* midgut progenitors do not produce metastatic tumours unless they also express *snail* (Christofi and Apidianakis, 2013; Campbell et al., 2019). Accordingly, we show that Rac1 promotes cell dissemination by inducing *snail*, in addition to actin cytoskeleton signalling genes, *Rok*, *sqh*, *Apr2*, and *Apr3*, and a positive feedback loop with the *grass-spz/spz5-Toll-cactus/dif/dorsal* pathway. Downregulation of core Toll signalling genes significantly decreases cell dissemination, with Toll receptor silencing almost abolishing it. Toll sufficiency was assessed by the expression of activated *spz* and *cactus^RNAi^*, each of which induced significant cell dissemination on its own. We used two *spz^RNAi^* transgenes, one of which did not inhibit sqh phosphorylation by *Ras^V12^*, while it inhibited suppression of DE-cadherin and induction of JNK phosphorylation. Likely, Toll signalling suppresses DE-cadherin and induces JNK phosphorylation directly or strongly, while it may induce sqh phosphorylation indirectly or less strongly.

Interestingly, FlyAtlas anatomical gene expression data show that *spz* is expressed in high levels in the hindgut compared to the midgut, suggesting that hindgut enterocytes might be primed for cell dissemination via the Toll pathway. Moreover, *spz5* participates in this network, as it can bind and induce Toll in addition to Toll-6, and both *spz* and *spz5* drive Toll-6-mediated JNK activation and cell migration in the context of organotropic metastasis (Mishra-Gorur et al., 2019; Chowdhury et al., 2019; Nonaka et al., 2018). Whether Spz and Spz5 ligands are released only by hindgut enterocytes or also by neighbouring or remote cells is yet to be determined.

In marked similarity with the *Ras^V12^* signalling network in hindgut enterocytes, *spz-Toll-dorsal-twist/snail* signalling and small Rho-GTPases operate during embryonic gastrulation (Rich and Glotzer, 2021) and culminate in the repression of DE-cadherin, a core component of adherens junctions (Bauer et al., 2006). Moreover, we find that *18w* interacts with *Ras^V12^* to induce cell dissemination, similarly to its synergism with Rho-GTPases in inducing apical constriction of salivary gland cells (Kolesnikov and Beckendorf, 2007). Of note, there are nine Toll receptors in *Drosophila*, and each may signal differently in different contexts, such as in border cell migration (Kleve et al., 2006), neuronal survival (Foldi et al., 2017), competition-induced cell death (Wu et al., 2015) and carcinogenesis (Mishra-Gorur et al., 2019; Ding et al., 2022). Since *18w/Toll2* affects cell dissemination without being part of the Toll pathway, additional Toll pathways may contribute to the *Ras^V12^* signalling network (Ding et al., 2022). Given the high expression of TLRs in multiple cancers (O'Neill, 2008; Zhang et al., 2009), and the conservation between Toll and TLR pathways (Gay and Gangloff, 2007; Gay et al., 2014), mammalian TLRs may have a similar role in sustaining oncogenic cell migration.

**Fig. 12.**
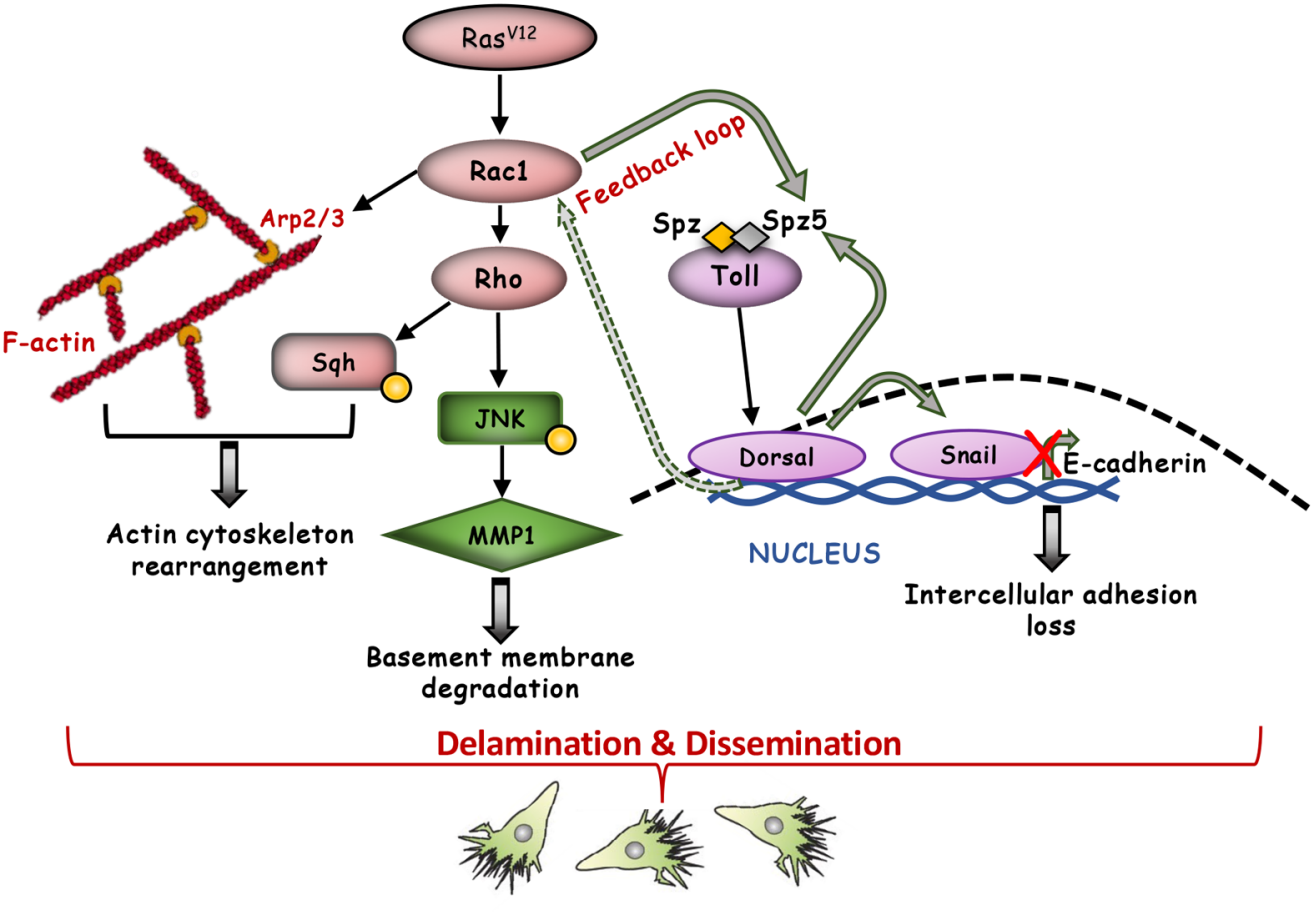
**Proposed *Ras^V12^* singling network leading to cell dissemination.** Our data support a signalling network in which Toll, JNK and actin cytoskeleton signalling genes, *Rac1*, *Rok*, *Sqh*, *Apr2* and *Apr3*, transmit the transformative *Ras^V12^* signalling that primes the hindgut enterocytes towards delamination and dissemination. *Ras^V12^* expression stimulates activation primarily of Rac1, which may in turn promote actin cytoskeleton rearrangements required for cell dissemination via Rho activation and Sqh phosphorylation. Moreover, Rac1 forms a positive feedback loop with the Toll immune pathway. Rac1 activated Toll drives the expression of *snail* and *spz5*, which in turn repress E-cadherin and sustain Toll signalling activation. Finally, Rac1 and Toll signalling converge on JNK activation and upregulation of *Mmp1* expression, leading to basement membrane degradation, allowing cells to invade neighbouring tissues.

Rho GTPase signalling (Brumby et al., 2011) and Toll signalling (Mishra-Gorur et al., 2019) are necessary in other contexts to induce JNK and invasive cell behaviour downstream of the Ras oncogene. Notwithstanding the possibility of JNK-independent activation of Mmp1 by Toll or Rac1, the simplest explanation to our findings is that Toll and Rac1 crosstalk and induce JNK and in turn Mmp1, because: (i) Toll and Rac1 signalling can activate each other, (ii) Toll or Rac1 signal can induce JNK, (iii) JNK can induce Mmp1, and (iv) Toll or Rac1 can induce Mmp1. In previous studies, bacterial infection and Imd signalling was shown to stimulate JNK-mediated Mmp1 expression in the hindgut facilitating cell dissemination by *Ras^V12^* (Bangi et al., 2012). Here, western blotting analysis indicates that activation of JNK by *Ras^V12^* is necessary but not sufficient for cell dissemination and is under the control of Rac1 and Toll signalling, which, in addition, oversee cytoskeletal rearrangements and DE-cadherin suppression (Fig. 12). In light of their conserved functions, inhibition of Toll and Rac1 may restrict Ras-induced cell overgrowth and tissue invasion in mammalian cells. It will, thus, be of interest to test their combinatorial inhibition against various types of Ras-activated tumours.

## MATERIALS AND METHODS

### Fly stocks

All fly stocks and crosses were maintained at 18°C on a 12:12 h light: dark cycle on standard fly food (agar, cornmeal, sucrose and Brewer's yeast, Tegosept and propionic acid). Adult female flies were collected shortly after eclosion and aged to 3-5 days old into fresh vials prior to each experiment. For temperature controlled hindgut enterocyte expression the *w;;byn-Gal4,tubGal80^ts^,UAS-gfp/TM6b* line was crossed to UAS lines: UAS-grassRNAi (Bl#51920), UAS-18wRNAi (Bl#30498), UAS-TollRNAi (VDRC_KK100078), UAS-spz5RNAi (Bl#67229), UAS-spzRNAi (Bl#34699), UAS-spzRNAi (VDRC_KK105017), UAS-SpzAct (gift by Petros Ligoxygakis), UAS-DorsalRNAi (VDRC_KK105491), UAS-DifRNAi (VDRC_KK100537), UAS-CactusRNAi (Bl#34784), UAS-CactusRNAi (Bl#34775), UAS-sqhRNAi (Bl#31542), UAS-sqhRNAi (Bl#33892), UAS-Rac1 (Bl#6291), UAS-RokCAT (Bl#6669), UAS-Rac1N17 (gift by Norbert Perrimon), UAS-sqhE20E21 (Bl#64411), UAS-Arp3RNAi (Bl#32921), UAS-RhoGEF (gift by Norbert Perrimon), UAS-Arp2RNAi/TM3,sb (Bl#27705), UAS-Arp3RNAi (VDRC_GD35260), UAS-HepAct (Bl#9306), yw; UAS-Lifeact-Ruby (Bl#35545), w; Byn-Gal4, tubGal80ts, UAS-gfp/TM6B (Bangi et al., 2012), w;UAS-RasV12 (Bl#64196). Crosses were reared at 18°C and adult flies were transferred to 29°C to induce the transgenes before experiments. We crossed females of the Gal4 line to UAS lines of interest using the reference line, *w^1118^*, as a basic control. Thus, half of the nuclear DNA, all the mitochondrial DNA, and the egg nutrients were supplied by mothers of the same genotype and were identical among the progeny compared. To alleviate concerns regarding remaining genetic background differences among the UAS lines and the reference line, we used alternative UAS lines per gene and many UAS lines per pathway in multiple instances.

### Dissemination assay

Per Bangi et al. (2012), anesthetized female flies were lined up and immobilized on glass slides coated with Vaseline with their ventral sides up. The glass slide was then transferred into a Petri dish and submerged in 1x PBS solution (10x PBS: 1.3 M NaCl, 0,07 M Na_2_HPO_4_, 0,03 M NaH_2_PO_4_), so that a complete coverage of the fly bodies was achieved. Using forceps, the abdomens were opened to make a ‘fillet’, intestines and ovaries removed and the number of GFP+ foci along the abdominal cavity was counted using a Leica M165 FC dissecting stereoscope with a GFP filter under a ×10 magnification. Each experiment was performed in duplicate (*n*=30 in each replicate). Error bars throughout represent standard deviation of the mean (s.d.). *P*-values were calculated using the Fisher's exact test with a 5×2 contingency table to assess the five phenotypic classes between two conditions at a time.

### Immunohistochemistry

Fifteen hindguts per genotype were dissected onto a silicon plate in 1x PBS drop and fixed for 1 h with 9% formaldehyde (FA) at room temperature (RT). For FasIII immunostaining, hindguts were fixed in 4% FA for 30 min at RT. Following completion of the fixation period the samples were rinsed three times with 1x PBS to remove any residual FA. Hindguts were then incubated into PBT (1× PBS, 0.5% BSA, 0.1% Triton X-100) blocking solution for 1 h to achieve tissue permeabilization and blocking of non-specific antibody binding sites. Then, blocking solution was removed and primary antibodies diluted in PBT solution were added and incubated with tissue samples overnight in the dark at 4°C. Primary antibodies: anti-Mmp1 antibody cocktail 1:100 (mouse, 3B8D12, 5H7H11 and 3A6B4 from DSHB), anti-Fas III 1:100 (mouse, 7G10 from DSHB). The next day, hindguts were washed three times for 10 min in PT (1x PBS, 0.2% Triton-X) solution. Secondary antibodies against mouse, rabbit or guinea pig conjugated to Alexa fluor 555 (Invitrogen) were used at 1:1000. Samples were incubated in secondary antibody solution including DAPI (Sigma, 1:3000 of 10 mg/ml stock) for 2 h at room temperature (RT), in the dark, with mild shaking. Finally, samples were washed three times with PT to remove unbound antibody and mounted on glass microscope slides in 20μl of Vectashield (Vector), covered with glass coverslips and sealed with nail polish. Images of the immunostained hindguts were captured using the LEICA TCS SP2 AOBS confocal microscope.

### Hindgut size

Length and width were assessed from GFP pictures acquired with a fluorescent Leica M165 FC stereomicroscope (at 6.3x magnification) and their width and length were analysed using ImageJ (https://imagej.net/ij/). Clicking on the Analyze, Set Scale option, using the length of the picture as 2048 pixels corresponding to 1.65 mm. Using the segmented line option, a line was drawn manually, starting from hindgut proliferating zone (HPZ) and ending just before the rectum. For the width measurement, straight lines indicating the width of pylorus (HPZ), the middle ileum and posterior ileum were manually drawn vertically to the gut length. The length of the lines was measured and thereby the gut's dimensions, by clicking on the Analyze, Measure option.

### Nuclei DNA content

Per previous studies (Tamamouna et al., 2020) nuclear DNA was calculated from non-saturated confocal images using ImageJ. Images of the middle hindgut were captured at 63× magnification, zoom 1× and 1024×1024 format and produced as a maximum projection of 10-15 sections serial imaging. Sum projections were used to measure the total DAPI fluorescence from individual nuclei using ImageJ in anterior and posterior images of the midguts. Specifically, each and every nucleus (excluding nuclei that overlapped) was selected manually using the circular selection tool of the software and surface and integrated density were acquired. Data points of hundreds of cells arising from ≥3 hindgut parts were added to generate BoxPlots.

### Reverse transcription quantitative PCR (RT-qPCR)

For RNA extraction 100 hindguts (without the rectum) were dissected for each genotype in three biological replicates. Tissue dissections were performed in 1x PBS drops on a silicon plate followed by an immediate freezing on dry ice, so that maximizing tissue integrity and eliminating potential RNA degradation. Sample tissues were homogenized at 50 Hz for 10 min in 500 μl Qiazol using the TyssueLyser LT (Qiagen) and a stainless-steel bead (5 mm diameter). Next, the homogenized tissues were incubated at RT for 5 min. Sample phase separation was mediated by the addition of 100 μl chloroform (CHCI_3_) followed by a vigorous shaking for 15-20 s and centrifugation at 12000 ***g*** for 15 min at 4°C. The resulting upper phase, which contains the RNA was carefully transferred into a clean Eppendorf tube. Finally, 250 μl isopropanol were added and mixed in the RNA sample and incubated at RT for 15 min. A second step of centrifugation at 12,000 ***g*** for 15 min at 4°C was performed to pellet the RNA. Supernatant was then discarded, and RNA pellet was carefully rinsed with 200 μl 70% EtOH. A last centrifugation step at 8000 ***g*** for 5 min at 4°C occurs to completely remove any residual EtOH. The pellet was then dissolved in 20 μl RNase free water and the concentration of RNA in the sample was determined using the NanoDrop 200c Spectrophotometer (Thermo Fisher Scientific).

For cDNA synthesis 800 ng of total RNA were used to synthesize the cDNA using Promega RQ1 RNase-Free DNase Kit according to the manufacturer’s protocol. RNA was treated with DNAse I mixture (RQ1 10× buffer, 1 μl RQ1 DNase enzyme from Promega) for 30 min at 37°C to eliminate the genomic DNA contamination. Reaction was ceased by the addition of RQ1 DNAse stop solution and 10 min incubation at 65°C. Reverse transcription was performed using 145.4 ng of the total DNAse-treated RNA by using the TaKaRa Prime ScriptTM RT Master Mix Kit. Following completion of the reverse transcription reaction, the cDNA is diluted (1:6) in RNase/DNase free water. For each qPCR reaction 6 μl cDNA, 4 μl gene specific primers and 10 μl KAPA SYBR Supermix (Kapa Biosystems) were used. qPCR amplification was then performed based on the following amplification program on the CFX96 Real-Time System/C1000 Thermal Cycler (Bio-Rad): 95°C for 30 s (initial denaturation), 40 cycles of 95°C for 10 s (denaturation), 60°C for 30 s (annealing), 65°C for 30 s (extension) and 65°C for 1 min (final extension). The expression of the genes of interest was normalized to the expression levels of two reference genes, *rpl32* and *α-tubulin* using the 2^−ΔΔCt^ method. Data were analysed using the Bio-Rad CFX Manager 3.1 program.

### Protein extraction

For sub-cellular protein fractionation hindgut tissues were collected on dry ice as described earlier. Tissue homogenization was performed at 50 Hz for 10 min into a 2 ml tube containing 150 μl Buffer A with 0.1% Triton (10 mM HEPES, 10 mM KCL, 1.5 mM MgCl2, 0.34 mM sucrose, 10% glycerol, 100 μl, 0,1% Triton-X 100, 1 mM DTT, 1 tablet of protease inhibitors, dissolved in DNAse-RNAse free water) and a stainless-steel bead using the TyssueLyser LT (Qiagen). Following homogenization, samples were incubated on ice for 30 min with vortexing every 10 min. 50 μl of the protein lysate were transferred into a clean tube and referred to as the whole tissue extract (WTE). The remaining 150 μl of protein extract were centrifuged at 21,000 ***g*** for 10 min at 4°C producing a supernatant (S1) and a pellet (P1). S1 sample was subjected to the centrifugation step as previously for four times until the S5 supernatant (cytoplasmic fraction-CF) was collected. Following completion of each centrifugation step, the supernatant was transferred into a clean tube while the pellet was discarded. P1 pellet was treated with 100 μl Buffer A (without Triton) and centrifuged at 6000 ***g*** for 10 min at 4°C. This step was performed twice to eliminate the cytoplasmic protein contamination of pellet and ensure purity. At the final centrifugation step supernatant is removed and P1 pellet is dissolved into 70 μl of Buffer B (10 mM HEPES, 3 mM EDTA, 0.2 mM EGTA, 1 mM DTT, 1 tablet of protease inhibitors, dissolved in DNAse-RNAse free water). The sample was then incubated on ice for 30 min with vortexing every 10 min. Completion of this step P1 denoted the nuclear protein fraction (NF) of the sample.

For western blotting the samples that were not subjected to sub-cellular fractionation were homogenized into Buffer A with 0.1% Triton followed by 30 min incubation on ice with vortexing every 10 min, as described above.

### Western blotting

Protein lysates were boiled in 4× Laemmli Buffer for 10 min at 95°C and separated using standard immunoblotting protocol. Small proteins (20-70 kDa) were separated through a 12% SDS–PAGE while larger proteins through an 8% SDS–PAGE. The gel was placed into an electrophoresis tank and run for 1-1.5 h in 200 V until efficient protein separation as indicated by the 240 kDa prestained protein ladder (Nippon Genetics) was achieved. Proteins were then transferred to a PVDF membrane, which had been previously soaked in methanol for 1 min. Protein transfer was carried out at 100 V for 1.5 h at 4°C. Next, the membrane was incubated in Ponceau S solution to confirm transfer efficiency, rinsed and blocked in 5% skimmed milk dissolved in 1× TBS-Tween 20 (TBS-T) buffer for 1 h on a shaking platform at RT. Then, the membrane was incubated with the primary antibody overnight at 4°C. Primary antibodies were diluted in 5% skimmed milk, 1x TBS-T, unless otherwise recommended. Primary antibodies: anti-E-cadherin 1:200 (rat, DCAD2 from DSHB), anti-sqh 1:5000 (rabbit, gift from Karess R.), anti-p-sqh 1:1000 (rabbit, 3671S from CST), anti-Mmp1 antibody cocktail 1:1000 (mouse, 3B8D12, 5H7H11 and 3A6B4 from DSHB). Following primary antibody incubation, the membrane was washed for 20 min with 1× TBS-T and then for additional 10 min with fresh 1× TBS-T. Finally, the membrane was incubated with an HRP-conjugated secondary antibody of choice (1:10,000 dilution) at RT for 1 h on a shaking platform. Then, the membrane was washed as before, and protein signal detection was performed using the SuperSignal™ West Femto Maximum Sensitivity Substrate (Invitrogen) and Syngene G-Box gel documentation system. Protein expression was quantified compared to the syntaxin (mouse, 1:1000, DSHB #8C3) loading control using the ImageJ software. The mean intensity of respective protein bands from three different immunoblots was used for the quantification.

### Transcriptomics analysis

RNA was isolated from three replicates of 100 control hindguts (*w;;byn-Gal4tubGal80^ts^UAS-gfp/+*) and hindguts expressing the *Ras^V12^* oncogene (*w;UAS-Ras^V12^/+;byn-Gal4tubGal80^ts^UAS-gfp/+*) for 7 days at 29°C. RNA quality was assessed on a Bioanalyzer (Agilent Technologies) with RNA 6000 Nano Kit reagents and protocol (Agilent Technologies), followed by use of the 3′ mRNA-Seq Library Prep Kit protocol for Ion Torrent (QuantSeq-LEXOGEN Vienna, Austria) according to the manufacturer's instructions. Briefly, up to 500 ng of RNA was used for first and second-strand synthesis, followed by amplification. Library quality and quantity were assessed on a Bioanalyzer with DNA High Sensitivity Kit reagents and protocol (Agilent Technologies). The libraries were pooled together and templated and enriched on an Ion Proton One Touch system. Templating was performed using the Ion PI Hi-Q OT-II 200 Kit (Thermo Fisher Scientific), followed by sequencing with the Ion PI Hi-Q Sequencing 200 Kit on Ion Proton PI V2 chips (Thermo Fisher Scientific) on an Ion Proton System, as per the manufacturer's instructions.

Raw reads were aligned to the *Drosophila* genomic build dm6 through in two steps. Firstly, reads were mapped with hisat2 (Kim et al., 2019) using the default parameters and then, the unmapped reads were mapped with bowtie2 (Langmead and Salzberg, 2012) using the local and very sensitive parameters. Prior to the statistical testing procedure, the gene read counts were filtered for possible artifacts that could affect the subsequent statistical testing procedures. Genes presenting any of the following were excluded from further analysis: (i) genes with zero reads (6531), (ii) genes with length less than 500 (817 genes), (iii) genes whose average reads per 100 bp was less than the 25th quantile of the total normalized distribution of average reads per 100 bp (824 genes with cutoff value 0.26135 average reads per 100 bp), (iv) genes with read counts below the median read counts of the total normalized count distribution (3931 genes with cutoff value) normalized read counts. The total (unified) number of genes excluded due to the application of all filters was 12055. The resulting gene counts were subjected to differential expression analysis for *byn-G4* versus *byn-G4 U-Ras^V12^* using the Bioconductor packagemetaseqR2 with the DESeq algorithm and the utr parameter (Fanidis and Moulos, 2021). DEGs were identified based on an absolute log2 fold change (|log2(FC)|>1) and a *P*-value <0.05. The code is publicly available at https://github.com/alex-galaras/panagi_et.al.2025. DEGs were further analysed using the DAVID (Database for Annotation, Visualization and Integrated Discovery) database to discover enriched functional-related gene groups. However, we manually curated the genes listed in three functional categories: cytoskeleton, immunity and stress, and cell polarity and adhesion, because of multiple false positive and false negative genes listed as cytoskeletal and immunity related.

## Supplementary Material

10.1242/biolopen.061960_sup1 Supplementary information
