# Supplementary material for: A feed-forward loop between Toll/NF-κB and Rac1 promotes epithelial to mesenchymal transition of Ras-oncogenic hindgut enterocytes in Drosophila
Source: Biol Open. 2025 Jun 17;14(6):bio061960. doi: 10.1242/bio.061960 (PMC12208402; doi:10.1242/bio.061960)
Supplement: Supplementary information [file biolopen-14-061960-s1.pdf]

Table S1. Cytoskeleton DEGs (Ras^{V12} vs Control)

Gene Name	Fold Change (log2)	Ratio	*p-value*	FDR
Act42A	2.79	6.90	1.3E-06	7.7E-05
Myo31DF	2.55	5.85	1.1E-05	4.9E-04
betaTub60D	2.48	5.59	8.7E-06	3.8E-04
rols	2.48	5.56	5.9E-05	2.0E-03
Arpc3A	2.13	4.39	5.5E-04	1.2E-02
Cortactin	2.12	4.36	2.8E-04	7.0E-03
capt	2.00	4.01	1.1E-03	2.1E-02
Fas2	1.96	3.90	1.6E-03	2.6E-02
Whamy	1.93	3.81	4.5E-03	5.8E-02
Akap200	1.89	3.71	1.5E-03	2.5E-02
trio	1.87	3.65	2.6E-03	3.9E-02
sqh	1.82	3.53	1.4E-03	2.4E-02
Fim	1.76	3.40	1.9E-03	3.0E-02
zip	1.76	3.39	3.7E-03	5.0E-02
ena	1.68	3.21	2.8E-03	4.0E-02
Sep2	1.66	3.16	1.6E-03	2.7E-02
twf	1.62	3.07	2.4E-03	3.6E-02
Pkn	1.61	3.06	3.8E-03	5.1E-02
mys	1.60	3.04	5.9E-03	6.8E-02
capu	1.54	2.90	1.6E-02	1.3E-01
Mlc-c	1.46	2.76	1.6E-02	1.3E-01
tsr	1.41	2.66	1.7E-02	1.4E-01
Pak	1.41	2.65	4.7E-02	2.6E-01
mst	1.40	2.64	2.2E-02	1.6E-01
Map205	1.39	2.62	1.7E-02	1.4E-01
Rok	1.34	2.54	2.2E-02	1.6E-01
stac	1.29	2.44	2.0E-02	1.5E-01
Jupiter	1.26	2.39	3.1E-02	2.0E-01
Arpc5	1.24	2.36	2.4E-02	1.7E-01
betaTub56D	1.20	2.30	3.6E-02	2.2E-01
Arpc1	1.17	2.26	2.8E-02	1.9E-01
Rep	1.15	2.22	4.1E-02	2.4E-01
Zasp52	-1.17	0.44	3.4E-02	2.2E-01
Mlc2	-1.35	0.39	2.0E-02	1.6E-01
Mhc	-1.42	0.37	1.7E-02	1.4E-01
sgg	-1.46	0.36	1.7E-02	1.4E-01
wupA	-1.57	0.34	5.0E-03	6.1E-02
spir	-1.61	0.33	1.9E-02	1.5E-01
Act87E	-1.69	0.31	5.2E-03	6.3E-02
Mlc1	-1.92	0.26	7.2E-04	1.4E-02
HERC2	-1.97	0.25	9.9E-03	9.8E-02
Myo28B1	-2.00	0.25	4.9E-03	6.1E-02
up	-2.00	0.25	7.0E-04	1.4E-02
corto	-2.03	0.24	4.6E-04	1.0E-02
Actn	-2.09	0.23	1.4E-04	4.2E-03
Tm2	-2.29	0.20	1.4E-04	4.1E-03
tau	-2.30	0.20	1.7E-04	4.8E-03

Biology Open • Supplementary information

Table S2. Immunity and Stress DEGs (Ras^{V12} vs Control).

[1]Imd pathway; [2]Toll pathway; [3]JNK/p38 pathway

Gene Name	Fold Change (log2)	Ratio	*p-value*	FDR
AttD[1]	8.09	272.67	2.1E-24	6.0E-21
AttB[1]	5.02	32.50	1.4E-12	4.5E-10
AttA[1]	4.75	26.83	3.8E-11	8.5E-09
PGRP-SA[2]	4.33	20.09	1.3E-11	3.3E-09
PGRP-SD[1]	4.16	17.83	2.5E-10	4.1E-08
Ets21C[3]	3.83	14.25	4.4E-06	2.1E-04
IM18	3.46	10.98	2.6E-08	2.7E-06
18w[2]	3.32	10.00	3.0E-06	1.5E-04
Rab30[3]	1.88	3.69	8.4E-03	8.7E-02
psh[2]	1.74	3.35	1.5E-02	1.3E-01
Dro[1]	1.68	3.21	6.6E-03	7.4E-02
grass[2]	1.67	3.18	8.9E-03	9.1E-02
Jra[3]	1.64	3.11	4.6E-03	5.9E-02
p38a[3]	1.48	2.79	2.4E-02	1.7E-01
l(2)34Fc[2]	1.36	2.57	1.6E-02	1.3E-01
key[1]	1.31	2.48	3.4E-02	2.2E-01
spirit[2]	1.19	2.28	4.9E-02	2.7E-01
IM3[2]	-1.42	0.37	7.4E-03	8.1E-02
IM4[2]	-1.53	0.35	2.3E-03	3.5E-02
sphe[2]	-1.88	0.27	3.3E-03	4.6E-02
sick[1]	-2.18	0.22	4.8E-04	1.0E-02
BaraA1[2]	-2.82	0.14	1.5E-06	8.7E-05
BaraA2[2]	-3.08	0.12	2.2E-06	1.2E-04

Table S3. Cell Polarity and Adhesion DEGs (Ras^{V12} vs Control) [1]Septate Junction; [2]Extracellular matrix; [3]Basement membrane

Gene Name	Fold Change (log2)	Ratio	*p-value*	FDR
Tig	4.75	26.98	2.2E-12	6.7E-10
Mec2	4.09	17.00	2.2E-06	1.2E-04
Tsp42Eb	3.42	10.74	9.9E-08	8.0E-06
bbg[1]	3.16	8.94	7.3E-07	4.8E-05
Invadolysin[2]	2.95	7.73	1.4E-06	8.4E-05
insc	2.81	7.00	8.8E-04	1.7E-02
Tsp2A[1]	2.59	6.03	2.5E-05	9.7E-04
mesh[1]	2.51	5.69	3.9E-05	1.4E-03
Tsp42Ed	2.49	5.61	5.6E-05	1.9E-03
Inx7	2.35	5.10	1.7E-03	2.8E-02
Ssk[1]	2.26	4.77	2.7E-04	6.9E-03
Mmp1[2]	2.10	4.30	2.0E-04	5.4E-03
Tsp42El	2.10	4.28	6.0E-04	1.3E-02
aPKC	2.03	4.07	2.5E-03	3.7E-02
scb	1.90	3.74	6.2E-04	1.3E-02
mew	1.78	3.42	2.8E-03	4.0E-02
pins	1.77	3.42	1.6E-02	1.3E-01
bark	1.70	3.25	3.0E-03	4.3E-02
pio	1.55	2.93	5.2E-03	6.3E-02
l(2)gl[1]	1.35	2.54	1.7E-02	1.4E-01
Tsp39D	1.18	2.26	4.6E-02	2.6E-01
Tsp42Ei	1.11	2.15	4.0E-02	2.4E-01
parvin	1.05	2.07	4.8E-02	2.6E-01
Col4a1[3]	-2.90	0.13	4.0E-06	2.0E-04
vkg[3]	-1.89	0.27	~0.05	2.7E-01

Biology Open • Supplementary information
